# 

**Published:** 1888-03-17

**Authors:** 


### SPECIAL NOTICE TO NEWSAGENTS, ADVERTISERS, AND THE TRADE.

### CHANGE OF OFFICE AND PUBLISHER.

Notice is hereby given that the Office of The Hospital will henceforth be at 38, Old Jewry (Cheapside end), E.C., to which address all communications should be sent. Mr. A. Doig has been appointed Advertising Agent, and Mr. J. H. S. Hanning, Manager. All Cheques and Post Office Orders should be made payable to J. H. S. Hanning, 38, Old Jewry, E.C. crossed Lloyds, Barnetts, and Bosanquets Bank (Limited).

### TO SUPERINTENDENTS, SECRETARIES, AND MATRONS.

The Editor will be glad to have a copy of the Regulations, and also of every Report issued from the Press of Asylums, Hospitals, Convalescent and Nursing Institutions, as well as those of other Charities, so soon as they are published and an early copy in duplicate of all appeals for funds. Notices of Annual and other Meetings, Festival Dinners, etc., will be welcomed. All such communications should be addressed to *The Editor, The Lodge, Porchester Square, London, W.* Any superintendent, secretary, matron, or other chief official who will regularly send such documents and information will be registered to receive free of cost a cover for binding The Hospital in half-yearly volumes on sending name and address to the publisher.

### AMUSEMENTS WITH PROFIT FOR ALL AGES.

**Rules.**—All answers must be addressed to the PRIZE EDITOR, THE HOSPITAL, 38, Old Jewry, Cheapside, London, E.C., not later than he *first* post on Thursday next. The full name and address must in all cases be given, but a *nom de plume* may be added for publication. Only one side of the paper must be written on.

### FOR MEN AND WOMEN.

**Rule.**—Competitors can enter for *all* quarterly competitions, but no competitor may take more than *two quarterly prizes* during the year.

### Acrostic Competition.

Second quarter commenced February 25th, 1888; ends May 19th, 1888.

A PRIZE of ONE GUINEA will be given to the competitor who gains the highest number of marks for best original acrostics during the ensuing quarter. All acrostics sent in for this competition will be credited according to merit, twelve marks being the highest score given for one acrostic.

A PRIZE of HALF-A-GUINEA to be given to the competitor who gains the highest score for solution of acrostics set. *One mark* only will be given to the competitors who solve all the lights of each acrostic.

Exception will be made in the case of *quotation acrostics*, when *two marks* will be given, *one* for the correct solution of all *lights*, and *one* mark for the *source* of the quotations.

This week credit will be given for

### No. 2. Original Double Acrostic.

Results of No. 1 Original Double Acrostic.—Patience (10), Brownie (9), Sister (8), Mater (8), A. M. E. (8), Gretta (8), Ellice (8), Reldas (8).

### Results of First Quarterly Original Acrostic Competition.

PASTEUR and BROWNIE having agreed to divide the prize of One Guinea, we have forwarded the sum of 10*s*. 6*d*. each to Pasteur (Rev. G. Moor, M.A., Worthing), Brownie (Rev. J. E. L. Nowers, 3, Comeragh Road, West Kensington, S.W.)

### Results of First Quarterly Test Acrostic Competition.

DUNCE and CONDORCET having also decided to divide the prize of half-a-guinea, the sum of 5*s*. 3*d*. has been forwarded to Dunce (Mrs. Young, 31, Merham Road, West Kensington, S.W.), and to Condorcet (Miss C. Montgomery, 26, Pembroke Gardens, W.)

N.B.—The above successful competitors are eligible to enter again for all quarterly competitions, but they will only be able to receive *one* more quarterly prize during the year.

### A) THE WORD COMPETITION.

Second Quarterly Competition commenced February 4th, 1888; ends April 28th, 1888.

Three prizes of one guinea, 15*s*. and 10*s*. 6*d*. will be given each quarter to the three competitors who obtain the highest number of marks; (1) by making the largest number of words derived from the word or phrase set for anatomical dissection; or (2) for the best answers to (B); or (3) by (1) and (2) combined.

We shall give the newspapers and periodicals for dissection during this quarter, that for this, the *seventh* week of the quarter, being

"CONTEMPORARY."

OBSERVE.—Proper names, abbreviations, foreign words, words of less than four letters, and repetitions are barred; plurals, and past and present participles, of verbs are allowed. Nuttall's *Standard* dictionary only to be used.

N.B.—All words sent in must be arranged *alphabetically*, with correct total affixed.

### Results of Fifth Week.

*The Echo*.– Gretta (6), Patience (6), Ellice (6), Sym (5), Dollie (5), E. E. (4), Nelle (4), Lancashire Witch (4), Lauriston (4), Lightowlers (4), Lodge (4), Brisbane (3), Condorcet (3), Butterfly (3), Dunce (3).

### (B) THE LITERARY COMPETITION.

Some people hate puzzles of all kinds, including word competitions, but they are fond of correspondence, and many sisters and mothers are continually receiving and sending letters to and from members of the family who are abroad in India or the Colonies. The constant coming and going between England and other portions of the British Empire make it a matter of interest and importance to know something about the life, the climate, the amusements, the adventures, the clothing, and the surroundings of those who seek a livelihood beyond the seas. We shall therefore try to find space for the publication of selected letters from abroad, which may be sent by any of our readers who have friends and relations in foreign lands. We shall give marks for these letters as well as for the word competitions which will count equally for these prizes, so that anyone may enter for both or either, the prizes to be given to those who get the highest number of marks.

### Letters from Across the Sea.

LIGHTOWLERS sends the following from a *Hospital Nurse* at

GRAHAMSTOWN, SOUTH AFRICA.

We were a cheerful party, fourteen of us, all going to mission work, but only myself came on to Grahamstown; the rest were bound for Bloemfontein, Kimberley, and other districts; we were all very sorry to part at the last, but I am happy in knowing that I have made friends in the colony, for we shall meet again no doubt. People are constantly moving in this country, and I hope to travel over some parts of it before I return to England. It is very interesting to see this land, it is so different from any other part of the world; the immense tracts of open country, long ranges of round hills for miles, with little valleys between, all bare of trees, with just a low growth of shrub or heather; you can see such a distance when you get on one end of the ranges. It is just like the wilderness of Judea, I believe, and makes one understand the history of the Israelites so much better than before. One can see the want of water and the shadeless burning sand, which would make them long for the shelter of the rock. This town lies in a hollow with hills all round, which have been planted with firs and gum trees; so there is shade and beauty, while the climate is lovely; quite cold now in winter, comparatively—that is to say, we are glad of a fire at night and warm clothing. In summer it is very hot, they tell me, but not so hot as further up at Kimberley. The vegetation is splendid: we have oranges and lemons hanging ripe on the trees now, and things grow wonderfully; maiden-hair ferns are wild in the little valleys in profusion, and the flowers this month are beyond description. In the summer everything gets too parched up; but now the weather is perfect. This home is a small sisterhood and an orphanage for girls, while we have two large schools as well. There are only three Sisters here, and the rest are workers—six in all, like myself, who came out for a year or two to teach. I am not in the schools myself, as I have been given district visiting, Sunday school class, with partial care of the orphans, of whom we have 17. So there is a great deal to be done for them. I have no nursing yet, but as soon as the hospital is opened at Port Elizabeth I shall be sent there. Occasionally I find I can help the sick folk in the district, and they send up for a nurse generally, even from the rich houses, when anybody is dying. So I have to keep in readiness. There is a dreadful want of work here among the poor, as we have none of the agencies there are in England to help, and the people are very bad. They are not really poor, as white people can always get work, and food is very cheap indeed; but our colonists are so irreligious, they seem to have left all their morals and religion in England, while the young who have been born here are not much better. The great work of the Church is to get the children and educate them to be real Christians, and for that it is that we need help in all parts of this great diocese. There is a demand for sisters from all quarters, which we cannot supply; but one of these Sisters has gone to Port Elizabeth to undertake parish work, and a school there; as that is a very important station, much larger in population than this, and most godless. We have a cathedral here and a regular staff of clergy, but they all work very hard, most of them teaching in the boys' school nearly all day, and going miles to out-lying districts for services on Sunday.

### Notices to Correspondents.

*K. M*—Many thanks for the touching lines, which we hope to insert at an early date. Contributions of this kind are always welcome when good.

### THE CHILDREN'S CORNER.

PRIZES.

FOR MOST CORRECT ANSWERS TO PUZZLES.

This Commenced October 1st, 1887. One Pound a Month.

A PRIZE OF THREE GUINEAS to the Competitor who shall have sent in the largest number of correct or best answers during the twelve months.

### Puzzles—Third Week.

No. 9. CHARADE. My *first* is in morning, my *second* in noon, my *third* in night, my *whole* makes a traveller's way pleasant.

No. 10. DOUBLE ACROSTIC.

Two British birds outshine the rest,
The *first* grass-green with scarlet crest,
In emerald and turquoise dressed,
The *last* with fiery-orange breast.

1. Though dark myself, centre of cheering rays,
2. A word that gives assent in Frenchmen's phrase.
3. Even from a Stoic's eye I draw a tear.
4. Northumbrian heroine—or you, my dear.
5. Three-cornered joy, to schoolboys dear.
6. Here David fled when foes were near.
7. An ancient king, famed for riches and power.
8. This term denotes tricks which dishonest are.
9. To make "multum *ex* parvo"—easy art.
10. Seize the oar, and do *your* part.

No. 11. CONUNDRUM. What is the difference between a cat and a comma?

No. 12. TRANSPOSITION.

A well-known tree transposed aright
Will bring to view a useful light.

### Results of First Week Puzzles.

No. 1. DOUBLE ACROSTIC.

S eawee D
N igge R
O hi O
W as P

No. 2. ENIGMATICAL LIST OF DISTINGUISHED PERSONS.—1. Wolf-e=Wolfe. 2. Shake-spear=Shakespear. 3. Cow-per=Cowper. 4. Marlborough=Marlborough.

No. 3. CHARADE.—Snow-ball=Snowball.

No. 4. Three ducks.

*All right*—A. C. K., Alsie, Excelsior, Plummy Fluff, Squeaker, Judy, Scrooge, Happy Eliza, Spider, Mount Edgcumbe, Jumps, Tyke, Bookworm, Gerald, H. Babette, Bagshot, Moina.—*Three right*—Umslopogaas, A. G. S., McCulloch. *N.B.*—Observe *new rule* No. VI.

### Conditions.

I. Every paper sent in must be clearly written, and one side only must be used. All competitors must mark at the head of their papers the number of their competition.

II. Each paper must be signed by the author with his or her real name and address. A *nom de plume* may be added, if the writer does not desire to be referred to by us by his real name. In the case of all prize-winners, however, the real name and address will be published.

III. All communications relating to prize competitions should be addressed to "The Prize Editor, THE HOSPITAL, 38, Old Jewry, London, E.C." Competitors are requested not to include in letters, etc., to the Prize Editor communications on matters of other business. All letters must be fully prepaid.

IV. The Prize Editor of "THE HOSPITAL" is the sole judge of the competitions, and his decision is in all cases final and without appeal.

V. The Prize Editor reserves the right to publish any paper sent in, whether it receives a prize or not. He does not hold himself responsible for any MSS. sent, nor can he undertake to return rejected competitions.

VI.—All children resident at school are requested to forward their *home* address in addition to the school one, when entering for the "Children's Corner" Competitions.

**Notice to all Correspondents.**—N.B.—All letters referring to this page, which do not arrive at 38, Old Jewry, Cheapside, London, E.C., by the *first post on Thursdays*, and are not addressed PRIZE EDITOR, will in future be disqualified and disregarded.

No one will be permitted to win the Monthly or Quarterly Prizes twice in any one year, the Annual prizes being offered especially to prevent this.

